# Analysis of factors influencing glucose tolerance in Japanese patients with non-alcoholic fatty liver disease

**DOI:** 10.1186/s13098-017-0264-7

**Published:** 2017-09-05

**Authors:** Satoko Ohmi, Masafumi Ono, Hiroshi Takata, Seiki Hirano, Shogo Funakoshi, Yuichi Nishi, Kumiko Yoshimura, Eri Amano, Yoshio Terada, Toshiji Saibara, Shimpei Fujimoto

**Affiliations:** 1 Department of Endocrinology, Metabolism, and Nephrology, Kochi Medical School, Kochi University, Kohasu, Oko-cho, Nankoku, Kochi 783-8505 Japan; 2 Gastroenterology and Hepatology, Kochi Medical School, Kochi University, Kohasu, Oko-cho, Nankoku, Kochi 783-8505 Japan

**Keywords:** Insulin secretion, Insulin sensitivity, Non-alcoholic fatty liver disease

## Abstract

**Background:**

While the association of the prevalence of non-alcoholic fatty liver disease (NAFLD) with impaired glucose metabolism has been reported, the factors influencing glucose tolerance in NAFLD remain to be clarified.

**Methods:**

Glucose tolerance of 131 Japanese patients diagnosed as NAFLD by histological findings of liver biopsy specimen was examined using 75 g-OGTT. According to Matteoni’s classification, patients were divided to 4 groups [M1 ~ 4, M1, 2: non-alcoholic fatty liver (NAFL); and M3, 4: non-alcoholic steatohepatitis (NASH)]. Based on the OGTT data, insulinogenic index (IGI) and QUICKI were calculated as indices of insulin secretion and insulin sensitivity, respectively. Plasma glucose 120 min after glucose loading (G_120_) was used as the index for glucose intolerance.

**Results:**

Stepwise multiple regression analysis using G_120_ as a dependent variable and log_e_-IGI, QUICKI, sex, BMI, age, NAFL/NASH as independent variables revealed that log_e_-IGI (β = −0.595) and QUICKI (β = −0.323) are significant factors predicting glucose intolerance (R^2^ = 0.403), indicating an important role of insulin secretion in glucose tolerance. These findings accord with glucose intolerance as high as 89.7% in patients with impaired insulin secretion defined by ≤43.2 pmol/mmol (40 μU/mg) IGI. Stepwise multiple regression analysis using QUICKI as a dependent variable and NAFL/NAFLD, sex, BMI, and age as independent variables revealed that BMI (β = −0.469) and NAFL/NAFLD (β = −0.204) are significant factors predicting insulin sensitivity (R^2^ = 0.248).

**Conclusion:**

Impairment of insulin secretion is the most important factor to predict glucose intolerance in NAFLD; severity of histological findings is associated with insulin sensitivity independent of adiposity in NAFLD.

## Background

The prevalence of nonalcoholic fatty liver disease (NAFLD) has been increasing worldwide. NAFLD is associated not only with liver-related morbidity and mortality, but also with an increased risk of cardiovascular disease [1]. NAFLD can be classified broadly into 2 subtypes: non-alcoholic fatty liver (NAFL), which is benign with negligible risk of progression to advanced fibrosis and liver-related mortality, and non-alcoholic steatohepatitis (NASH), which has substantial risk of progression to advanced fibrosis and liver-related mortality. NAFLD is closely related to metabolic disorders including obesity, glucose intolerance, hyperinsulinemia, and dyslipidemia. Many cohort studies showed that NAFLD was independently associated with the incidence of diabetes, which causes microangiopathy and is an important risk factor of cardiovascular disease [1]. In addition, previous studies showed that diabetes was an independent predictor of progression to fibrosis in NASH patients [2, 3] and that glycemic control may prevent histological progression of NAFLD [4].

While the association of the prevalence of NAFLD with impaired glucose metabolism has been reported, the association between the severity of NAFLD and glucose tolerance remains to be clarified. While impaired insulin secretion and insulin resistance are the main pathophysiological mechanisms of type 2 diabetes [5, 6], little is known of the relative contribution of these factors to glucose intolerance in patients with NAFLD. In the present study, these issues were investigated using oral glucose tolerance test (OGTT) in Japanese patients with NAFLD.

## Methods

### Patients

We analyzed pooled data of 134 Japanese patients in whom 75-g OGTT was performed, and a liver biopsy was carried out to determine histological severity of NAFLD between 2006 and 2015 in a usual clinical situation at Kochi University Hospital, Japan. Informed consent was obtained from each patient. In all patients, the current and past daily alcohol intake was less than 20 g per day; details regarding alcohol consumption were obtained independently by at least two physicians and were confirmed by close family members. None of patients took medications that could cause NAFLD including methotrexate, tamoxifen and corticosteroids. Patients with the following disorders were excluded: secondary causes of steatohepatitis, drug-induced liver disease, alcoholic liver disease, viral hepatitis, autoimmune hepatitis, primary biliary cirrhosis, α1-antitrypsin deficiency, hemochromatosis, Wilson’s disease, and biliary obstruction. Among the patients, 127 patients did not take any antidiabetic medication including oral hypoglycemic agents, insulin and GLP-1 receptor agonist and 7 patients took oral hypoglycemic agents (nateglinide, n = 2; voglibose, n = 2; pioglitazone, n = 2; metformin, n = 1). We included patients treated with nateglinide and voglibose, but excluded those treated with pioglitazone and metformin in analysis, considering the possible remaining efficacy of the agents during OGTT. Finally, data of 131 patients were analyzed. The study protocol was approved by the Ethical Review Board of Kochi Medical School.

### Laboratory examination

75-g OGTT was performed in the morning after overnight fast in patients less than a week before liver biopsy. In patients taking nateglinide and voglibose, medication was stopped for OGTT. Samples were drawn just before and at 30, 60, 90 and 120 min after ingestion of glucose. Plasma glucose was measured by the glucose oxidase method. Plasma immunoreactive insulin (IRI) was measured using sandwich ELISA (Access^®^ Ultrasensitive Insulin, Beckman Coulter, Brea, CA). HbA1c was measured by HPLC. Using the 2006 World Health Organization (WHO) criteria [7], subjects were categorized as having normal glucose tolerance (NGT) [fasting plasma glucose (FPG) < 6.1 mmol/L and 2-h plasma glucose (2-h PG) < 7.8 mmol/L], impaired glucose metabolism (IGM) [either impaired fasting glucose (IFG): FPG ≥ 6.1 and <7.0 mmol/L and/or impaired glucose tolerance (IGT): 2-h PG ≥ 7.8 and <11.1 mmol/L) or diabetes (DM) (FPG ≥ 7.0 mmol/L and/or 2-h PG ≥ 11.1 mmol/L). To convert insulin units to SI units, conversion factor (1 U = 6.00 nmol) was used instead of previous factor (1 U = 7.174 nmol), which is determined according to potency of a new standard of human insulin established by the World Health Organization in 1987 [8] and adopted by the American Diabetes Association [9].

### Indices of insulin secretion and insulin sensitivity/resistance

In 75-g OGTT, plasma glucose (G) and IRI levels (I) at fasting (G_0_, I_0_), 30 (G_30_, I_30_), 60 (G_60_, I_60_), 90 (G_90_, I_90_) and 120 min (G_120_, I_120_) after glucose loading were determined, respectively. G_m_ and I_m_ were calculated by dividing Area Under Curve of G and I during OGTT by 120 min, respectively. As insulin secretion indices, insulinogenic index (IGI) and HOMA-β were calculated using the following formula [10, 11]: IGI: [I_30_ − I_0_ (pmol/L)]/[G_30_ − G_0_ (mmol/L)]; HOMA-β: I_0_ (μU/mL) × 20/[G_0_ (mmol/L) − 3.5]. As an insulin resistance index, HOMA-R was calculated using the following formula^9^: I_0_ (μU/mL) × G_0_ (mmol/L)/22.5. As insulin sensitivity indices, QUICKI and Matsuda index were calculated using the following formula [12, 13]: QUICKI: 1/[log_10_ G_0_ (mg/dL) + log_10_ I_0_ (μU/mL)]; Matsuda index: 10,000/[G_0_ (mg/dL) × I_0_ (μU/mL) × G_m_ (mg/dL) × I_m_ (μU/mL)]^0.5^. Oral disposition index (DI_O_) was calculated using the following formula [14]: IGI/I_0_ (pmol/L).

### Pathology

Patients enrolled in this study underwent percutaneous liver biopsy under ultrasonic guidance after obtaining informed consent. Formalin-fixed, paraffin-embedded liver sections were stained routinely with hematoxylin–eosin, silver reticulin, and Masson trichrome. All of the specimens were examined by an experienced pathologist who was unaware of the clinical and biochemical data of the patients. Histological diagnosis for NAFLD was performed according to the methods of Matteoni et al. [15]. Histological findings were classified to non-alcoholic fatty liver (NAFL) without (type 1, M1) or with (type 2, M2) inflammation and to non-alcoholic steatohepatitis (NASH) without (type 3, M3) or with (type 4, M4) fibrosis.

### Statistical analysis

Statistical analysis was performed with the StatView 5.0 system (SAS institute Inc., Cary, NC). Normally distributed continuous data are presented as mean ± SE and non-normally distributed continuous data are presented as median value, 25th percentile value and 75th percentile value. Difference among more than three groups was determined by analysis of variance (ANOVA) for normally distributed continuous data, by Kruskal–Wallis tests for non-normally distributed continuous data, and Scheffe’s test was performed as post hoc analysis. Difference of dichotomous and categorical data among groups was determined by χ^2^ tests. For cut-off values to divide into low or normal IGI group and normal or high HOMA-R groups, normal values originally defined by the Japan Diabetes Society and confirmed as optimal cut-off points to predict incidence of diabetes in Japanese population [IGI: 43.2 pmol/mmol (40 μU/mg); HOMA-R: 1.6] [16] were used. The relationship between the parametric data and between the nonparametric data was determined by Pearson analysis and by Spearman analysis, respectively. Stepwise multiple regression analysis was performed in which normally-distributed log_e_-transformed IGI, log_e_-transformed DIo, and log_e_-transformed Matsuda index were used. *P* values <0.05 were considered statistically significant.

## Results

### Clinical, biochemical and pathological profiles of groups according to glucose tolerance

Age, sex, HbA1c, glucose levels during OGTT, and indices of insulin secretion (IGI, HOMA-β) were significantly different among NGT, IGM, and DM groups (Table 1). BMI and IRI levels during OGTT were not significantly different among 3 groups. Indices of insulin sensitivity/resistance including HOMA-R and Matsuda index were not different among 3 groups. QUICKI was marginally different among 3 groups, but post hoc analysis revealed no significant difference. DIo, a measure of β-cell function adjusted for insulin sensitivity, which is a good predictor of diabetes [14], differed among 3 groups. The prevalence of NASH was significantly higher in IGM and DM groups compared to that in NGT group.

**Table 1:** Clinical, biochemical, and pathological profiles of NGT, IGM and DM groups

	NGT	IGM	DM	*P* value
n	47	51	33	
Age	38.9 ± 2.5	48.0 ± 2.1*	54.7 ± 2.4*	<0.0001
Sex (M/F)	33/14	28/23	12/21*	0.0110
BMI (kg/m^2^)	29.7 ± 0.9	28.7 ± 0.7	28.4 ± 0.8	0.5028
HbA1c (%)	5.08 ± 0.08	5.24 ± 0.06	6.46 ± 0.18*,**	<0.0001
G_0_ (mmol/L)	5.13 ± 0.09	5.54 ± 0.08	7.02 ± 0.33*,**	<0.0001
G_120_ (mmol/L)	6.90 ± 0.24	9.08 ± 0.21*	14.86 ± 0.66*,**	<0.0001
G_m_ (mmol/L)	7.95 ± 0.22	9.62 ± 0.17*	13.35 ± 0.48*,**	<0.0001
I_0_ (pmol/L)	77.9 ± 7.9	74.3 ± 6.7	86.9 ± 11.9	0.6009
I_120_ (pmol/L)	541.8 ± 58.7	702.1 ± 68.3	681.8 ± 90.8	0.2035
I_m_ (pmol/L)	534.1 ± 46.1	547.1 ± 53.7	411.2 ± 44.5	0.1520
IGI (pmol/mmol)	124.4 (71.5, 191.6)	75.2* (39.9, 116.6)	29.7*,** (15.8, 54.8)	<0.0001
HOMA-β	144 (84, 206)	92 (62, 180)	72* (53, 115)	0.0006
HOMA-R	2.43 (1.51, 3.70)	2.41 (1.55, 4.21)	3.66 (2.05, 5.52)	0.0508
QUICKI	0.339 ± 0.005	0.336 ± 0.005	0.320 ± 0.006	0.0427
Matsuda index	2.89 (2.08, 5.20)	2.74 (1.61, 4.49)	2.31 (1.46, 3.44)	0.1561
DI_O_ (mmol/L^−1^)	1.72 (1.33, 3.70)	1.15* (0.75, 1.79)	0.42*,** (0.23, 0.74)	<0.0001
M1 n(%)	9 (19.1)	7 (13.7)	1 (3.0)	0.0093***
M2 n(%)	19 (40.4)	9 (17.7)	10 (30.3)	
M3 n(%)	6 (12.8)	10 (19.6)	6 (18.2)	
M4 n(%)	13 (27.7)	25 (49.0)	16 (48.5)	

G_0_, G_120_ and G_m_ are plasma glucose (PG) levels at 0 (fasting) and 120 min after glucose loading and mean PG level in 75 g OGTT, respectively. I_0_, I_120_ and I_m_ are serum IRI levels at 0 (fasting) and 120 min after glucose loading and mean IRI level in 75 g OGTT, respectively. G_m_ and I_m_ are calculated by dividing Area Under Curve of PG and IRI during OGTT by 120 min, respectively. IGI: insulinogenic index

M1, M2, M3, and M4: type 1, 2, 3, and 4 in histological classification of NAFLD by Mateonni, respectively

* *P* < 0.05 vs. NGT

** *P* < 0.05 vs. IGM

*** NAFL (M1 + M2) vs. NASH (M3 + M4) among NGT, IGM, and DM groups; *P* < 0.05, NGT vs. IGM; *P* < 0.05, NGT vs. DM

### Glucose tolerance and pathological finding in groups according to indices of insulin secretion and resistance

Subjects were divided to 2 groups according to the cut-off value of IGI (low-IGI vs. normal-IGI) or HOMA-R (normal-R vs. high-R). Prevalence of NGT and DM was lower and higher in low-IGI compared to that in normal-IGI, respectively (Table 2). Glucose intolerance was as high as 89.7% in low-IGI, but it was 53.3% in normal-IGI. Prevalence of DM was higher in high-R compared to that in normal-R, but prevalence of NGT did not differ. Prevalence of NASH was not different between low-IGI and high-IGI, but was higher in high-R group compared to that in low-R group.

**Table 2:** Glucose tolerance and histological classification in groups according to IGI and HOMA-R

	n (%)	Low-IGI 39 (100.0)	Normal-IGI 92 (100.0)	P	Normal-R 35 (100.0)	High-R 96 (100.0)	P
Glucose tolerance							
NGT	n (%)	4 (10.3)	43 (46.7)	<0.0001* <0.0001**	16 (45.7)	31 (33.3)	0.1564* 0.0284**
IGM	n (%)	14 (35.9)	37 (40.2)		15 (42.9)	36 (37.5)	
DM	n (%)	21 (53.8)	12 (13.1)		4 (11.4)	29 (30.2)	
Histological classification							
M1	n (%)	5 (12.8)	12 (13.0)	0.5947***	9 (25.7)	8 (8.3)	0.0338***
M2	n (%)	10 (25.6)	28 (30.5)		11 (31.4)	27 (28.1)	
M3	n (%)	10 (25.6)	12 (13.0)		6 (17.2)	16 (16.7)	
M4	n (%)	14 (36.0)	40 (43.5)		9 (25.7)	45 (46.9)	

M1, M2, M3, and M4: type 1, 2, 3, and 4 in histological classification of NAFLD by Mateonni, respectively

*IGI* insulinogenic index, *low-IGI* IGI ≤ 43.2 pmol/mmol (40 μU/mg), *normal-IGI* IGI > 43.2 pmol/mmol (40 μU/mg), *normal-R* HOMR-R ≤ 1.6, *high-R* HOMA-R > 1.6

* NGT vs. IGM + DM

** NGT + IGM vs. DM

*** NAFL (M1 + M2) vs. NASH (M3 + M4)

### Clinical, biochemical and pathological profiles of groups according to histological classification

Age and HbA1c were different, but sex, BMI, and glucose and IRI levels during OGTT did not differ among 4 groups (Table 3). Indices of insulin secretion including IGI and HOMA-β were not different, but indices of insulin sensitivity/resistance except Matsuda index differed among 4 groups. DIo was lower in M2, M3, and M4 compared to that in M1. Prevalence of NGT was lower in M4 compared to M1 and M2, but the prevalence of DM did not differ among 4 groups.

**Table 3:** Clinical profiles of groups according to histological classification of NAFLD by Mateonni

	M1	M2	M3	M4	P value
n	17	38	22	54	
Age	39.4 ± 3.1	41.3 ± 2.8	51.9 ± 3.6	50.0 ± 2.1	0.0033
Sex (M/F)	12/5	22/16	12/10	27/27	0.5159
BMI (kg/m^2^)	28.5 ± 1.5	29.8 ± 0.8	26.8 ± 0.9	29.4 ± 0.7	0.1418
HbA1c (%)	4.99 ± 0.10	5.39 ± 0.13	5.99 ± 0.30*	5.55 ± 0.11	0.0070
G_0_ (mmol/L)	5.16 ± 0.11	5.69 ± 0.15	6.12 ± 0.36	5.87 ± 0.20	0.1305
G_120_ (mmol/L)	8.01 ± 0.53	9.35 ± 0.59	10.80 ± 1.10	10.17 ± 0.51	0.1068
G_m_ (mmol/L)	8.88 ± 0.30	9.58 ± 0.38	10.68 ± 0.75	10.27 ± 0.40	0.1348
I_0_ (pmol/L)	48.7 ± 9.4	81.3 ± 8.5	74.4 ± 10.4	88.3 ± 8.6	0.0797
I_120_ (pmol/L)	568.0 ± 134.9	638.9 ± 77.9	536.4 ± 72.7	704.4 ± 65.6	0.4840
I_m_ (pmol/L)	516.3 ± 84.5	493.9 ± 55.6	395.6 ± 49.5	561.6 ± 48.0	0.2663
IGI (pmol/mmol)	87.6 (32.9, 170.2)	67.9 (39.0, 126.3)	48.7 (35.8, 76.4)	89.4 (43.0, 130.4)	0.1965
HOMA-β	70 (51, 104)	125 (62, 169)	82 (54, 153)	109 (76, 189)	0.1645
HOMA-R	1.58 (1.10, 1.85)	3.41 (1.53, 4.11)	2.43 (1.31, 5.39)	3.045 (1.87, 5.16)	0.0049
QUICKI	0.363 ± 0.010	0.330 ± 0.005	0.333 ± 0.008	0.326 ± 0.004*	0.0014
Matsuda index	3.20 (2.69, 6.26)	2.88 (1.87, 4.97)	2.79 (1.65, 5.65)	2.65 (1.38, 3.44)	0.0845
DI_O_ (mmol/L^−1^)	2.42 (1.14, 3.81)	1.10* (0.70, 1.59)	1.03* (0.39, 1.45)	1.22* (0.56, 2.08)	0.0061
OGTT					
NGT n(%)	9 (52.9)	19 (50.0)	6 (27.2)	13 (24.1)	0.0241** 0.2610***
IGM n(%)	7 (41.2)	9 (23.7)	10 (45.5)	25 (46.3)	
DM n(%)	1 (5.9)	10 (26.3)	6 (27.2)	16 (29.6)	

G_0_, G_120_ and G_m_ are plasma glucose (PG) levels at 0 (fasting) and 120 min after glucose loading and mean PG level in 75 g OGTT, respectively. I_0_, I_120_ and I_m_ are serum IRI levels at 0 (fasting) and 120 min after glucose loading and mean IRI level in 75 g OGTT, respectively. G_m_ and I_m_ are calculated by dividing Area Under Curve of PG and IRI during OGTT by 120 min, respectively. IGI: insulinogenic index

* *P* < 0.05 vs. M1

** NGT vs. IGM + DM among M1, M2, M3 and M4 groups; *P* < 0.05, M1 vs. M4; *P* < 0.05, M2 vs. M4

*** NGT + IGM vs. DM among M1, M2, M3 and M4 groups

### Analysis to identify predictive factors for glucose intolerance

In simple correlation, log_e_-IGI and log_e_-DIo, and QUICKI and log_e_-Matsuda index were strongly correlated; BMI and age, BMI and QUICKI, log_e_-DIo and QUICKI, and BMI and log_e_-Matsuda index were moderately correlated (Table 4). As indices for glucose intolerance, HbA1c and G_120_ were used. In simple correlation, HbA1c and log_e_-IGI, HbA1c and log_e_-DIo, G_120_ and log_e_-IGI, and G_120_ and log_e_-DIo were moderately correlated (Table 5). In multiple regression models to predict HbA1c or G_120_, age, BMI and NAFL/NASH were not selected as significant contributors by stepwise examination, but indices of insulin secretion (log_e_-IGI or log_e_-DIo) and indices of insulin sensitivity (QUICKI or log_e_-Matsuda index) were significant determinants (Table 6). Standard coefficients of log_e_-IGI were larger than that of QUICKI or log_e_-Matsuda index in models (model 1, 2, 3). In addition, DIo was a dominant determinant for G_120_ in model 4. Taking these findings together, impairment of insulin secretion is the most important factor to predict glucose intolerance in NAFLD.

**Table 4:** Simple correlation between possible determinants of HbA1c and G_120_

	Age	Sex	BMI	Log_e_-IGI	Log_e_-DI_O_	QUICKI	Log_e_-MI	FL/SH
Age	1.000							
Sex	−0.355	1.000						
BMI	−0.400	0.110	1.000					
Log_e_-IGI	−0.391	0.219	0.262	1.000				
Log_e_-DI_O_	−0.293	0.087	−0.050	0.821	1.000			
QUICKI	0.052	−0.157	−0.454	−0.143	0.444	1.000		
Log_e_-MI	0.057	−0.131	−0.495	−0.269	0.295	0.909	1.000	
FL/SH	0.244	−0.104	−0.074	−0.033	−0.167	−0.124	−0.120	1.000

*Sex* male = 1, female = 0; *FL/SH* NAFL = 0, NASH = 1; *log*_*e*_ *-IGI* log_e_-transformed insulinogenic index; *log*_*e*_ *-DIo* log_e_-transformed oral disposition index; *log*_*e*_ *-MI* log_e_-transformed Matsuda index

**Table 5:** Coefficients in simple correlation between HbA1c or G_120_ and possible determinants

	HbA1c	G_120_
Age	0.292	0.282
Sex	−0.161	−0.160
BMI	−0.014	−0.152
Log_e_-IGI	−0.588	−0.603
Log_e_-DIo	−0.656	−0.639
QUICKI	−0.319	−0.276
Log_e_-MI	−0.200	n.d.
FL/SH	0.224	0.172

*Sex* male = 1, female = 0; *FL/SH* NAFL = 0, NASH = 1; *log*_*e*_ *-IGI* log_e_-transformed insulinogenic index; *log*_*e*_ *-DIo* log_e_-transformed oral disposition index; *log*_*e*_ *-MI* log_e_-transformed Matsuda index; *n.d.* not determined since G_120_ is used to calculate Matsuda index

**Table 6:** Multiple regressions for HbA1c and G_120_

Dependent variable	Independent variables	Coef.	Std. coef.	F value	R^2^ (|R|)
HbA1c (Model 1^a^)	QUICKI Log_e_-IGI	−8.416 −0.476	−0.365 −0.632	28.6 85.8	0.477 (0.691)
HbA1c (Model 2^b^)	Log_e_-MI Log_e_-IGI	−0.443 −0.512	−0.358 −0.679	25.6 92.1	0.466 (0.683)
G_120_ (Model 3^c^)	QUICKI Log_e_-IGI	−3.302 −0.202	−0.323 −0.595	21.8 73.8	0.403 (0.635)
G_120_ (Model 4^d^)	Sex Log_e_-DIo	−1.050 −2.134	−0.144 −0.626	4.5 86.4	0.428 (0.655)

*Sex* male = 1, female = 0; *NAFL/NASH* NAFL = 0, NASH = 1; *log*_*e*_ *-IGI* log_e_-transformed insulinogenic index; *log*_*e*_ *-MI* log_e_-transformed Matsuda index; *log*_*e*_ *-DIo* log_e_-transformed oral disposition index

^a^Age, sex, BMI and NAFL/NASH were not significant determinants in this model

^b^Age, sex, BMI and NAFL/NASH were not significant determinants in this model

^c^Age, sex, BMI and NAFL/NASH were not significant determinants in this model

^d^Age, BMI, QUICKI and NAFL/NASH were not significant determinants in this model

### Analysis to identify predictive factors for indices of insulin sensitivity and insulin secretion

In multiple regression models to predict QUICKI or log_e_-Matsuda index, NAFL/NASH was a significant determinant independent of BMI (Table 7). However, NAFL/NASH was not a significant contributor to predict log_e_-IGI and log_e_-DIo, which significantly correlate with age.

**Table 7:** Multiple regressions for indices of insulin sensitivity and insulin secretion

Dependent variable	Independent variables	Coef.	Std. coef.	F value	R^2^ (|R|)
QUICKI (Model 1^a^)	BMI NAFL/NASH	−0.003 −0.476	−0.469 −0.204	37.2 7.0	0.248 (0.498)
Log_e_-MI (Model 2^b^)	BMI NAFL/NASH	−0.063 −0.245	−0.508 −0.186	45.6 6.1	0.279 (0.528)
Log_e_-IGI (Model 3^c^)	Age	−0.025	−0.391	23.1	0.147 (0.391)
Log_e_-DIo (Model 4^d^)	Age BMI	−0.024 −0.040	−0.373 −0.199	16.8 4.8	0.119 (0.345)

*Log*_*e*_ *-IGI* log_e_-transformed insulinogenic index; *log*_*e*_ *-MI* log_e_-transformed Matsuda index; *log*_*e*_ *-DIo* log_e_-transformed oral disposition index; *sex* male = 1, female = 0; *NAFL/NASH* NAFL = 0, NASH = 1

^a^Age and sex were not significant determinants in this model

^b^Age and sex were not significant determinants in this model

^c^Sex, BMI and NAFL/NASH were not significant determinants in this model

^d^Sex and NAFL/NASH were not significant determinants in this model

## Discussion

In NGT group in the present study, average BMI was around 30 kg/m^2^, median IGI was around 150 pmol/mmol, and median HOMA-R was around 2.5, which were higher than the values in the general Japanese NGT population (average BMI: around 22 kg/m^2^; median IGI: around 95 pmol/mmol; median HOMA-R: around 0.9) [16], indicating that the NAFLD patients in the present study are obese, hyperinsulinemic, and insulin-resistant compared to the normal Japanese population. Indeed, in 131 patients in the present study, 101 (77%) patients had BMI ≥25 kg/m^2^ and were defined as obese by Japanese criteria [17], much higher than the prevalence in the general Japanese population (around 25%) [18]. These results are compatible with the strong association between NAFLD and obesity [19–21].

We show that impairment of insulin secretion is the most important factor to predict glucose intolerance in NAFLD. Glucose intolerance as high as 89.7% is found in patients with impaired insulin secretion defined by <43.2 pmol/mmol (40 μU/mg) IGI. In simple correlation, indices of hyperglycemia moderately correlate with index of insulin secretion, but weakly correlate with indices of insulin sensitivity. In multiple regressions to predict hypeglycemia, standard coefficients of the index of insulin secretion were larger than those of insulin sensitivity. These results suggest an important role of insulin secretion to compensate against reduced insulin sensitivity to maintain glucose homeostasis in patients with NAFLD. These results are compatible with the longitudinal observation based on data from obese Pima Indians regarding transition from NGT to diabetes by Weyer C et al. [22]. They proposed that progressors and non-progressors to diabetes differ essentially in that progressors cannot compensate by increased insulin secretion for the increased demand due to increased insulin resistance; non-progressors are those who can maintain normoglycemia by increased insulin secretion. Insulin secretion continues to decrease during deterioration of glucose tolerance in progressors; insulin secretion continues to increase in non-progressors who keep NGT despite increased insulin resistance. Affection of NAFLD on the relationship between insulin secretion and insulin sensitivity remain to be fully elucidated. There is only one report on this issue, in which comparison between obese children with NAFLD diagnosed by ultrasonography and matched children without NAFLD revealed no affection of NAFLD on the relationship between insulin secretion and insulin sensitivity [23]. These results are compatible with the result in the present study that histological severity of NAFLD was not a significant contributor to predict DIo, an index of insulin secretion adjusted for insulin sensitivity.

Several reports indicate that indices of insulin secretion are lower in patients with NASH compared to those in BMI-matched control [24–26]. However, there are few reports regarding affection of histological severity of NAFLD on insulin secretion, although hypoadiponectinemia caused by histological severity of NASH may predict impairment of insulin secretion in nondiabetic nonobese patients with NASH [27]. C-peptide-genic index, a similar index to IGI, is not significantly reduced in patients with NASH compared to that in patients with NAFL [25], which is compatible with the results that IGI did not differ among groups according to histological classification (Table 3) and that IGI was not correlated with histological severity of NAFLD (Tables 4, 7). On the other hand, DIo significantly differed among groups according to histological classification (Table 3), which is compatible with the result in previous study that the β-cell index, another index of insulin secretion adjusted for insulin sensitivity, was significantly lower with histological severity [26]. We speculate that these results are derived from confounding effects, since histological severity was not a significant factor to predict DIo in multivariate analysis (Table 7).

In patients with NAFLD, insulin sensitivity is reduced compared to that in control subjects [19, 28–30]. One of the reasons for reduced insulin sensitivity in NAFLD is adiposity; patients with NAFLD have higher BMI than that of control subjects [28, 31]. However, insulin sensitivity in patients with NAFLD is still reduced compared to obese subjects without NAFLD matched for BMI [29, 30, 32]. This suggests that factors other than adiposity may contribute to insulin resistance in NAFLD, which is compatible with our present finding that the severity of histological findings affects insulin sensitivity in NAFLD independently of BMI in multiple regression analysis. In previous reports, histological severity in NAFLD was significantly correlated with insulin sensitivity/resistance indices, but these results were not necessarily adjusted with BMI [33–35]. Our present results also accord with a recent report that severity of histological findings correlates with insulin sensitivity after adjustment for BMI [36]. The mechanism of correlation between severity of histological findings and insulin sensitivity independent of whole body adiposity remains to be elucidated. One possibility is that accumulation of some kinds of lipids other than triglyceride in liver may cause hepatic insulin resistance [37, 38]. Another possibility is that histological severity may involve mitochondrial abnormality, whole body oxidation, and secretion of hepatokines that affect insulin sensitivity of liver and whole body [36, 39].

In the meta-analysis comparing NGT individuals of African, Caucasian, and East Asian ethnicity, those of African ethnicity had the lowest insulin sensitivity and the highest insulin secretion, and those of East Asian ethnicity had the highest insulin sensitivity and the lowest insulin secretion, among the 3 subpopulations [40]. Interestingly, a hyperbolic relationship between insulin sensitivity and insulin secretion in African, Caucasian, and East Asian ethnicity was confirmed in the NGT, and the product of insulin sensitivity and insulin resistance (disposition index) decreased as glucose intolerance progressed to IGM and to DM in all 3 subpopulations [40]. These results suggest that ethnicity affects insulin sensitivity and insulin secretion to a greater extent, but affects the disposition index to a lesser extent. Although insulin sensitivity in American patients with NASH was more reduced compared to the present study [26], little is known about the effect of ethnicity on insulin sensitivity, insulin secretion, and on the disposition index in individuals with NAFLD. A further meta-analytical study is necessary to answer these questions.

A follow-up liver biopsy revealed that diabetes is an important risk factor for fibrosis progression in NAFLD [41, 42]. In addition, a decrease in HbA1c is strongly associated with improvement of fibrosis in NAFLD [4]. These results suggest that predicting progression of glucose intolerance in NAFLD is important to prevent a histological progression of NAFLD. The results of the present study may contribute to discriminating high-risk group of diabetes from low-risk group, and may be useful for early diabetes detection and intervention for individuals with NAFLD.

### Limitations

The present study has several limitations. First, insulin sensitivity was not measured by hyperinsulinemic euglycemic clamp, the gold standard method. Second, since this study is cross-sectional, a causal association cannot be evaluated. Longitudinal study to find factors to predict progression of glucose intolerance is necessary. Third, this study may be affected by selection bias and may not represent the whole population, since data were collected in a single institute in Japan. Further multicenter collaborative research is needed.

## Conclusion

This study establishes that impairment of insulin secretion is the most important factor to predict glucose intolerance in Japanese patients with NAFLD. In addition, the severity of histological findings affects insulin sensitivity independent of adiposity in NAFLD.

## Abbreviations

NAFLD: nonalcoholic fatty liver disease
NAFL: non-alcoholic fatty liver
NASH: non-alcoholic steatohepatitis
OGTT: oral glucose tolerance test
GLP-1: glucagon like peptide-1
IRI: immunoreactive insulin
ELISA: enzyme-linked immunosorbent assay
HbA1c: glycated hemoglobin
WHO: World Health Organization
NGT: normal glucose tolerance
IGM: impaired glucose metabolism
IFG: impaired fasting glucose
IGT: impaired glucose tolerance
FPG: fasting plasma glucose
2-h PG: 2-h plasma glucose
G: glucose
I: IRI
IGI: insulinogenic index
HOMA: homeostasis model assessment
QUICKI: quantitative insulin sensitivity check index
DI_O_: Oral disposition index
ANOVA: analysis of variance
BMI: body mass index

## References

[1] Anstee QM, Targher G, Day CP (2013). Progression of NAFLD to diabetes mellitus, cardiovascular disease or cirrhosis. Nat Rev Gastroenterol Hepatol..

[2] Angulo P, Keach JC, Batts KP, Lindor KD (1999). Independent predictors of liver fibrosis in patients with nonalcoholic steatohepatitis. Hepatology.

[3] Nakahara T, Hyogo H, Yoneda M, Sumida Y, Eguchi Y, Fujii H (2014). Japan Study Group of Nonalcoholic Fatty Liver Disease. Type 2 diabetes mellitus is associated with the fibrosis severity in patients with nonalcoholic fatty liver disease in a large retrospective cohort of Japanese patients. J Gastroenterol.

[4] Hamaguchi E, Takamura T, Sakurai M, Mizukoshi E, Zen Y, Takeshita Y (2010). Histological course of nonalcoholic fatty liver disease in Japanese patients: tight glycemic control, rather than weight reduction, ameliorates liver fibrosis. Diabetes Care.

[5] Kahn SE (2003). The relative contributions of insulin resistance and beta-cell dysfunction to the pathophysiology of Type 2 diabetes. Diabetologia.

[6] Martin BC, Warram JH, Krolewski AS, Bergman RN, Soeldner JS, Kahn CR (1992). Role of glucose and insulin resistance in development of type 2 diabetes mellitus: results of a 25-year follow-up study. Lancet.

[7] World Health Organization (2006). Definition and diagnosis of diabetes mellitus and intermediate hyperglycemia: report of a WHO/IDF Consultation.

[8] Vølund A (1993). Conversion of insulin units to SI units. Am J Clin Nutr.

[9] American Diabetes Association. Diabetes. Système International Units for Plasma, Serum or Blood Concentrations. http://diabetes.diabetesjournals.org/site/misc/SIunits.pdf.

[10] Seltzer HS, Allen EW, Herron AL, Brennan MT (1967). Insulin secretion in response to glycemic stimulus: relation of delayed initial release to carbohydrate intolerance in mild diabetes mellitus. J Clin Invest.

[11] Matthews DR, Hosker JP, Rudenski AS, Naylor BA, Treacher DF, Turner RC (1985). Homeostasis model assessment: insulin resistance and β-cell function from fasting plasma glucose and insulin concentrations in man. Diabetologia.

[12] Katz A, Nambi SS, Mather K, Baron AD, Follmann DA, Sullivan G (2000). Quantitative insulin sensitivity check index: a simple, accurate method for assessing insulin sensitivity in humans. J Clin Endocrinol Metab.

[13] Matsuda M, DeFronzo RA (1999). Insulin sensitivity indices obtained from oral glucose tolerance testing: comparison with the euglycemic insulin clamp. Diabetes Care.

[14] Utzschneider KM, Prigeon RL, Faulenbach MV, Tong J, Carr DB, Boyko EJ (2009). Oral disposition index predicts the development of future diabetes above and beyond fasting and 2-h glucose levels. Diabetes Care.

[15] Matteoni CA, Younossi ZM, Gramlich T, Boparai N, Liu YC, McCullough AJ (1999). Nonalcoholic fatty liver disease: a spectrum of clinical and pathological severity. Gastroenterology.

[16] Morimoto A, Tatsumi Y, Deura K, Mizuno S, Ohno Y, Miyamatsu N (2013). Impact of impaired insulin secretion and insulin resistance on the incidence of type 2 diabetes mellitus in a Japanese population: the Saku study. Diabetologia.

[17] Matsuzawa Y, Inoue S, Ikeda Y, Sakata T, Satito Y, Sato Y (2000). New criteria for diagnosis of obesity. J Jpn Soc Intern Med..

[18] The Japanese Ministry of Health, Labour and Welfare: National Health and Nutrition Survey; 2011.

[19] Bellentani S, Bedogni G, Miglioli L, Tiribelli C (2004). The epidemiology of fatty liver. Eur J Gastroenterol Hepatol.

[20] Chalasani N, Younossi Z, Lavine JE, Diehl AM, Brunt EM, Cusi K (2012). The diagnosis and management of non-alcoholic fatty liver disease: practice guideline by the American Gastroenterological Association, American Association for the Study of Liver Diseases, and American College of Gastroenterology. Gastroenterology.

[21] Dietrich P, Hellerbrand C (2014). Non-alcoholic fatty liver disease, obesity and the metabolic syndrome. Best Pract Res Clin Gastroenterol.

[22] Weyer C, Bogardus C, Mott DM, Pratley RE (1999). The natural history of insulin secretory dysfunction and insulin resistance in the pathogenesis of type 2 diabetes mellitus. J Clin Investig.

[23] Bedogni G, Mari A, De Col A, Marazzi N, Tiribelli C, Manco M (2016). Nonalcoholic fatty liver is not associated with the relationship between insulin secretion and insulin sensitivity in obese children: matched case-control study. Child Obes..

[24] Musso G, Gambino R, Cassader M (2010). Lipoprotein metabolism mediates the association of MTP polymorphism with beta-cell dysfunction in healthy subjects and in nondiabetic normolipidemic patients with nonalcoholic steatohepatitis. J Nutr Biochem.

[25] Musso G, Cassader M, De Michieli F, Rosina F, Orlandi F, Gambino R (2012). Nonalcoholic steatohepatitis versus steatosis: adipose tissue insulin resistance and dysfunctional response to fat ingestion predict liver injury and altered glucose and lipoprotein metabolism. Hepatology.

[26] Siddiqui MS, Cheang KL, Luketic VA, Boyett S, Idowu MO, Patidar K (2015). Nonalcoholic steatohepatitis (NASH) is associated with a decline in pancreatic beta cell (β-cell) function. Dig Dis Sci.

[27] Musso G, Gambino R, Biroli G, Carello M, Fagà E, Pacini G (2005). Hypoadiponectinemia predicts the severity of hepatic fibrosis and pancreatic beta-cell dysfunction in nondiabetic nonobese patients with nonalcoholic steatohepatitis. Am J Gastroenterol.

[28] Marchesini G, Brizi M, Morselli-Labate AM, Bianchi G, Bugianesi E, McCullough AJ (1999). Association of nonalcoholic fatty liver disease with insulin resistance. Am J Med.

[29] Chalasani N, Deeg MA, Persohn S, Crabb DW (2003). Metabolic and anthropometric evaluation of insulin resistance in nondiabetic patients with nonalcoholic steatohepatitis. Am J Gastroenterol.

[30] Qureshi K, Clements RH, Saeed F, Abrams GA (2010). Comparative evaluation of whole body and hepatic insulin resistance using indices from oral glucose tolerance test in morbidly obese subjects with nonalcoholic fatty liver disease. J Obes.

[31] Salgado AL (2010). Carvalho Ld, Oliveira AC, Santos VN, Vieira JG, Parise ER. Insulin resistance index (HOMA-IR) in the differentiation of patients with non-alcoholic fatty liver disease and healthy individuals. Arq Gastroenterol.

[32] Ortiz-Lopez C, Lomonaco R, Orsak B, Finch J, Chang Z, Kochunov VG (2012). Prevalence of prediabetes and diabetes and metabolic profile of patients with nonalcoholic fatty liver disease (NAFLD). Diabetes Care.

[33] Dixon JB, Bhathal PS, O’Brien PE (2001). Nonalcoholic fatty liver disease: predictors of nonalcoholic steatohepatitis and liver fibrosis in the severely obese. Gastroenterology.

[34] Schwimmer JB, Deutsch R, Rauch JB, Behling C, Newbury R, Lavine JE (2003). Obesity, insulin resistance, and other clinicopathological correlates of pediatric nonalcoholic fatty liver disease. J Pediatr.

[35] Nakamura A, Yoneda M, Fujita K, Tajima K, Kikuchi K, Nakajima A (2011). Impact of glucose tolerance on the severity of non-alcoholic steatohepatitis. J Diabetes Investig.

[36] Kato K, Takeshita Y, Misu H, Zen Y, Kaneko S, Takamura T (2015). Liver steatosis is associated with insulin resistance in skeletal muscle rather than in the liver in Japanese patients with non-alcoholic fatty liver disease. J Diabetes Investig.

[37] Farese RV, Zechner R, Newgard CB, Walther TC (2012). The problem of establishing relationships between hepatic steatosis and hepatic insulin resistance. Cell Metab.

[38] Matsuzaka T, Shimano H (2011). Molecular mechanisms involved in hepatic steatosis and insulin resistance. J Diabetes Investig.

[39] Sanyal AJ, Campbell-Sargent C, Mirshahi F, Rizzo WB, Contos MJ, Sterling RK (2001). Nonalcoholic steatohepatitis: association of insulin resistance and mitochondrial abnormalities. Gastroenterology.

[40] Kodama K, Tojjar D, Yamada S, Toda K, Patel CJ, Butte AJ (2013). Ethnic differences in the relationship between insulin sensitivity and insulin response: a systematic review and meta-analysis. Diabetes Care.

[41] Adams LA, Sanderson S, Lindor KD, Angulo P (2005). The histological course of nonalcoholic fatty liver disease: a longitudinal study of 103 patients with sequential liver biopsies. J Hepatol.

[42] McPherson S, Hardy T, Henderson E, Burt AD, Day CP, Anstee QM (2015). Evidence of NAFLD progression from steatosis to fibrosing-steatohepatitis using paired biopsies: implications for prognosis and clinical management. J Hepatol.

